# P-2087. Clinical Application of Broad-Range PCR in Pediatric Patients

**DOI:** 10.1093/ofid/ofae631.2243

**Published:** 2025-01-29

**Authors:** Christina Gagliardo, Katia Camille Halabi, David Singer, Sarah Qurbanali, Maria Davydova, Aspasia Katragkou

**Affiliations:** Atlantic Health System/Thomas Jefferson University, Morristown, New Jersey; Atlantic Health System, Morristown, New Jersey; Atlantic Health System: Goryeb Children's Hospital, Basking Ridge, New Jersey; Morristown Medical Center, Morristown, New Jersey; Morristown Medical Center, Morristown, New Jersey; Goryeb Children's Hospital, Atlantic Health System, Morristown, New Jersey

## Abstract

**Background:**

Broad-range PCR (BRPCR) and next-generation sequencing (NGS) have the potential to be useful adjunctive tools in diagnosing the infectious etiology of a wide range of pediatric infections. We aimed to assess the clinical utility and application of BRPCR and NGS in children with suspected infections.

**Figure:**
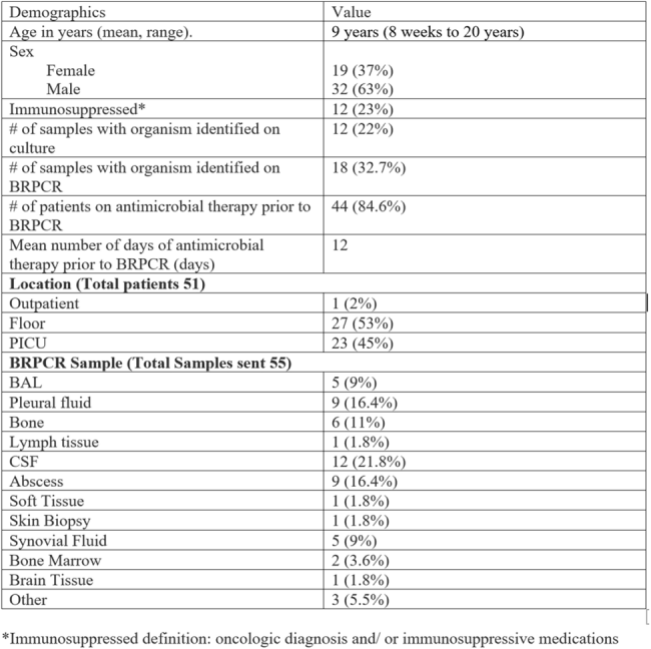

**Methods:**

A retrospective chart review was performed on pediatric patients with clinical samples sent for BRPCR (University of Washington Molecular Microbiology Laboratory, Seattle, WA, USA) between January 2023 and December 2023. Data was extracted from the electronic medical record, and descriptive statistics were performed.

**Results:**

51 patients had 55 samples sent for BRPCR. The demographics and sample information are listed in Table 1. A total of 18 (35.3%) patients had an organism identified on culture or BRPCR that was contributory to the clinical picture. Of those, 4 (7.3%) were identified on culture but not on BRPCR, and 6 (11%) were identified on BRPCR but not on culture. 4 (7.3%) cases identified the same organism on culture and BRPCR, while 4 (7.3%) cases identified different organisms on culture and concomitant BRPCR. In 6 (11.8%) cases, antibiotics were de-escalated, and in 7 (13.7%) cases, antibiotics were discontinued.

**Conclusion:**

BRPCR can be a valuable adjunct to identifying causative organisms in children with suspected infections and can help tailor antibiotic management. Our results highlight the potential impact of BRPCR results to reduce broad-spectrum antibiotic use. Further studies are needed to guide the optimal use of BRPCR and determine its clinical impact on management of pediatric infections.

**Disclosures:**

All Authors: No reported disclosures

